# Cross-sectional Survey of Hypertension Management among Patients Stratified by Chronic Kidney Disease in Japan

**DOI:** 10.31662/jmaj.2025-0037

**Published:** 2025-08-01

**Authors:** Kazuo Kobayashi, Keiichi Chin, Takayuki Furuki, Hiroyuki Sakai, Masaaki Miyakawa, Kei Asayama, Narumi Eguchi, Tomohiro Katsuya, Kazuyoshi Sato, Kouichi Tamura, Akira Kanamori

**Affiliations:** 1 Committee of Hypertension and Kidney Disease, Kanagawa Physicians Association, Yokohama, Japan; 2 Department of Medical Science and Cardiorenal Medicine, Yokohama City University Graduate School of Medicine, Kanagawa, Japan; 3 Japan Medical Association, Tokyo, Japan; 4 Department of Hygiene and Public Health, Teikyo University School of Medicine, Tokyo, Japan; 5 Japan Medical Association Research Institute, Tokyo, Japan; 6 Katsuya Clinic, Hyogo, Japan

**Keywords:** achievement rate, chronic kidney disease, hypertension paradox, target blood pressure

## Abstract

**Introduction:**

Despite progress in hypertension management, blood pressure (BP) control remains insufficient, and this so-called hypertension paradox is an urgent issue. Following the 2019 revision of the Guidelines for Hypertension Management, we reported that patients requiring stringent BP regulation highlight the unachieved goals of hypertension management. BP management is a basic and validated strategy for patients with chronic kidney disease (CKD) but its achievement rate has been under-reported.

**Methods:**

We collected and compared cross-sectional data of patients with hypertension in Kanagawa, Japan for 2011 and 2014 by accessing the Japan Medical Association Database of Clinical Medicine.

**Results:**

Patients with and without CKD were included as follows: 316/488 in 2011, 152/946 in 2014, and 396/385 in 2021, respectively. In the 2021 study, the target office BP (130/80 mmHg) and home BP (<125/75 mmHg) were achieved in 36.3% and 57.9% of patients with CKD, respectively, and this was a significant improvement over the results reported in the 2014 study (p < 0.05). In contrast, the 2021 study on patients without CKD had a significantly lower achievement rate for a stringent BP target compared to the 2014 study (p < 0.05), especially for patients without diabetes (p = 0.004). Unlike trends in patients without CKD, home BP control in patients with hypertension and CKD has improved over the past decade.

**Conclusions:**

The stringent BP target achievement rate remains insufficient in patients with CKD, indicating that CKD campaigns or resolution of clinical inertia resulting from an insufficient number of concomitant drugs is warranted.

## Introduction

Among the common lifestyle-related diseases, hypertension has the highest prevalence with 1.28 billion cases worldwide and 43 million patients in Japan thought to have hypertension ^(1), (2)^. However, 33% are unaware that they have hypertension, 56% are treated, and, among patients with hypertension, only 27% show well-controlled blood pressure (BP) ^(1)^. This status is termed the hypertension paradox and is an urgent issue that needs to be addressed worldwide. After the Systolic Blood Pressure Intervention Trial (SPRINT trial) ^(3)^ that revealed the superiority of strict BP management was reported, the Japanese Guidelines for the Management of Hypertension were revised in 2019 (JSH2019) ^(1)^, and more stringent BP targets have been imposed on patients compared to previous guidelines.

To evaluate the reality of the management of hypertension in clinical practice in Kanagawa prefecture, Japan, the Committee on Hypertension and Kidney Disease of the Kanagawa Physicians Association reported cross-sectional studies in 2008 ^(4)^, 2011 ^(5)^, and 2014 ^(6)^. Following the revision of the JSH2019 guidelines, we also conducted the 2021 study in cooperation with the Japan Medical Association Database of Clinical Medicine (J-DOME) which was founded in 2018 to improve the level of medical management for diabetes and hypertension. In our 2021 study, an insufficient control of BP was observed in patients who are required to exercise stringent BP control ^(7)^. In particular, low achievement rates (17.9% for an office BP target of <130/80 mmHg and 20.6% for a home BP target of <125/75 mmHg) were observed in patients whose BP target had been made more stringent after the JSH2019 revised guidelines, including patients with hypertension who did not have chronic kidney disease (CKD) or diabetes. The small number of antihypertensive drugs (1.7 drugs) made it difficult to achieve a BP target, and clinical inertia may correlate with these results in clinical practice.

CKD is recognized as an independent factor for end-stage kidney disease and also cardiovascular disease (CVD) ^(8)^. Hypertension and CKD progression are correlated ^(9)^ and the management of BP is one of the most basic strategies for patients with CKD and BP ^(10)^. In Japan, the low rate of achieving BP target goals for patients with CKD was reported in 2011 ^(11)^. Several campaigns for promoting awareness about CKD have been conducted, and CKD guidebooks or guidelines ^(10), (12)^ have been published to advance CKD management by general practitioners (GPs). In 2018, official documentation was reported by the Japanese Ministry of Health, Labor, and Welfare (Tokyo, Japan) that aimed at promoting measures against the spread of CKD (https://www.mhlw.go.jp/stf/shingi2/0000172968_00002.html). CKD awareness is now spreading in Japan; however, very little research has been conducted regarding BP management of patients with CKD.

This study aimed to clarify the state of BP management of patients with CKD compared to those without CKD using the data from our cross-sectional hypertension reports (the 2011, 2014, and 2021 studies).

## Materials and Methods

### Participants of this study

This is a post hoc analysis of our 2021 report, and details of the study participants and statistical methods employed have been described previously ^(7)^.

In brief, our 2021 study data were accessed from the J-DOME database that had initiated to register patients with diabetes and hypertension in January 2018 and July 2020, respectively. Over 19,000 participants with diabetes, hypertension, or both who gave verbal informed consent had been registered from 486 medical facilities in Japan in the J-DOME database up to December 2022. The criteria for including patients in the 2021 study were described as follows: 1) patients with hypertension; 2) patients who consulted GPs regularly or certified diabetes or hypertension specialists in medical hospitals or clinics located in Kanagawa prefecture; 3) the registration periods were between September 2021 and March 2022; and 4) patients’ age was 20 years or more. Patients without hypertension or younger than 20 years were excluded. Based on these criteria, in our 2021 study, the data of 835 patients from 26 hospitals or clinics located in Kanagawa prefecture were anonymized and used. In this CKD subgroup analysis, patients were divided into two categories, the CKD (+) and CKD (-) groups, using the CKD diagnostic criteria defined by the Kidney Disease Outcomes Quality Initiative (K/DOQI) clinical practice guidelines ^(13)^. We excluded 49 patients whose detailed proteinuria or albuminuria data could not be ascertained. Consequently, 385 patients in the CKD (+) and 396 patients in the CKD (−) groups were evaluated in the 2021 study.

The details of our 2011 and 2014 reports have been published ^(5), (6)^. In brief, these studies were conducted by the Kanagawa Physicians Association in October 2011 and from October to November 2014. For participant enrollment, anonymized registration forms including clinical data were mailed to the member of the Kanagawa Physicians Association. The details of the selection of study participants are shown in Supplementary Figure 1. The numbers of patients in the CKD (+) and CKD (−) groups were 316/488 in the 2011 study and 152/946 in the 2014 study, respectively. These earlier studies have also been included in the post-hoc analysis contained in the present study. 1

Clinical data that include sex, age, height, body weight (BW), BP in the office, and use of antihypertensive drugs were involved in this study. We also collected data on history of hypertension, smoking, drinking, complications (diabetes, CKD, ischemic heart disease, and cerebral vascular disease), BP at home, pulse rate, glycated hemoglobin A_1c_, estimated glomerular filtration rate (eGFR), urine albumin-to-creatinine, qualitative proteinuria, and estimated daily amount of salt intake. The following formula was used to calculate the eGFR value:

eGFR (mL/min/1.73 m^2^) = 194 × age^-0.287^ × serum creatinine^-1.094^ × (0.739 for women) ^(14)^.

Employing the K/DOQI clinical practice guidelines for CKD ^(13)^, patients with CKD were extracted from the 2011 study. However, since eGFR data were not collected in the 2014 study, patients were categorized as having CKD at the discretion of the attending GPs at the time of registration. From the data of spot urine samples, the estimated daily amount of salt intake was calculated using Tanaka’s formula ^(15)^.

### Blood pressure in the office and at-home measurements

A validated cuff-oscillometric device was used for the measurement of office BP at each medical facility. According to the JSH2019 guidelines ^(1)^, the measurement of BP in the office should be performed in a quiet situation after allowing the patient to rest for a few minutes while sitting with uncrossed legs. Therefore, the first BP reading was used in the subsequent analysis.

Home BP was measured according to the JSH2019 guidelines ^(1)^. Patients were instructed to measure their BP at home in a sitting position in an appropriate environment after resting for 1-2 minutes, with their legs not crossed, and with the position of the upper arm cuffs maintained at the level of the heart. Patients were instructed to measure their home BP once or twice in the morning. The mean values during the week just before the consult with the medical facility were recorded for the database. Oscillometric devices with upper arm cuffs are widely used in Japan for home BP monitoring.

The JSH2019 guidelines set the office and home BP target values based on the characteristics or complications of patients. Regarding the BP target values in this analysis, the 2011, 2014, and 2021 studies follow the JSH2009, JSH2014, and JSH2019 guidelines, respectively (Supplementary Figure 2). Supplementary Figure 3 shows the distribution of study participants depending on the target BP values in each study.

### Statistical analysis

For all statistical analyses, we used IBM SPSS Statistics software (version 28.0; IBM Inc., Armonk, NY, USA). Covariates with normal distribution are described as mean ± standard deviation (SD), whereas covariates that showed skewed distributions are described as median (interquartile range; 25th percentiles, 75th percentiles). For the comparison between the two groups, differences in parametric data and non-parametric data were analyzed by the unpaired t-test and Mann-Whitney rank-sum test, respectively. For the comparison of differences in proportions, the chi-square test was performed. Analysis of variance was used for the comparison of differences in continuous values among three groups. If the significance was confirmed by a p-value of less than 0.05, a Bonferroni correction was used as a post-hoc test.

To calculate the predicted BP values, the multiple linear regression analysis was performed with the values of sex, age, body mass index (BMI), alcohol drinking, smoking, and diabetes as covariates. With coefficients for each covariate, adjusted BP values were calculated.

### Ethics statement

This survey was performed in accordance with the principles of the Declaration of Helsinki. The Ethics Committee of the Kanagawa Prefecture Medical Association, Japan approved this study (Ethical Approval Number: KREC2204; July 29, 2022). The Ethical Approval of J-DOME was also obtained through the ethics review board of the Japan Medical Association (Ethical Approval Number: 28-3; the latest approval was provided on February 17, 2023).

## Results

### Clinical characteristics, use of antihypertensive drugs, and blood pressure levels among the three studies

The characteristics and details of prescribed antihypertensive drugs of patients with or without CKD in each study year are shown in Table 1a and 1b, respectively.

**Table 1.** Characteristics and Details of Antihypertensive Drugs of Patients in Each Study Year.

Study year	2011	2014	2021	P-value^♯^
**a. Patients in CKD(+) group**				
Number of cases	316	152	396	
Sex (women)	156 (49%)^†^	57 (38%)^*^	165 (42%)	0.03
Age (years-old)	73.4 ± 10.7	71.7 ± 12.5	72.9 ± 10.2	0.26
BW (kg)	60.7 ± 12.3^‡^	61.6 ± 11.8^‡^	65.8 ± 13.0^*†^	<0.001^§^
BMI	24.4 ± 3.6^‡^	24.2 ± 4.7^‡^	25.3 ± 3.6^*†^	0.002
History of hypertension (years)	9.2 ± 7.3	no data	13.7 ± 8.5	<0.001
	(n = 286)		(n = 237)	
Current smoker	59 (25%)	34 (33%)	43 (11%)	<0.001
	(n = 241)^‡^	(n = 102)^‡^	(n = 396)^*†^	
Current drinker	93 (41%)	36 (44%)	168 (43%)	0.86
	(n = 227)	(n = 82)	(n = 392)	
DM	64 (20%)^†‡^	52 (34%)^*‡^	292 (74%)^*†^	<0.001
IHD	9 (3%)^†‡^	24 (16%)^*^	57 (15%)	<0.001
			(n = 389)^*^	
CVD	23 (7%)	15 (10%)	36 (9%)	0.53
			(n = 385)	
eGFR(mL/min/1.73 m^2^)	58.6 ± 18.7	no data	54.8 ± 17.0	0.004
	(n = 313)		(n = 395)	
Presence of proteinuria or albuminuria	157 (52%)	112 (74%)	135 (37%)	0.001
	(n = 302)^†‡^	(n = 152)^*‡^	(n = 369)^*†^	
Estimated Salt intake (g/day)	no data	no data	8.9 ± 2.0	
			(n = 108)	
Presence of the guidance of salt restriction diet	261 (83%)	no data	269 (76%)	0.04
Numbers of antihypertensive drugs	2.0 ± 1.0^†‡^	2.3 ± 1.0^*‡^	1.8 ± 0.9^*†^	<0.001
The prescription of antihypertensive drugs				
CCB	233 (74%)	124 (82%)	284 (72%)	0.06
ARB	241 (76%)^‡^	121 (80%)^‡^	258 (65%)^*†^	<0.001
ACEi	10 (3%)	8 (4%)	18 (5%)	0.64
MRB	17 (5%)	12 (8%)	36 (9%)	0.17
β-blocker	47 (15%)	28 (18%)	50 (13%)	0.22
α-blocker	28 (9%)^‡^	19 (13%)^‡^	13 (3%)^*†^	<0.001
Diuretics	44 (14%)^†^	38 (25%)^*‡^	39 (10%)^†^	<0.001
Thiazide	42 (13%)	28 (18%)	no data	0.15
Loop	2 (1%)	10 (7%)	no data	<0.001

**b. Patients in CKD (-) group**				
Number of cases	488	946	385	
Sex (women)	251(51%)	506 (54%)^‡^	176 (46%)^†^	0.04
Age (years-old)	66.8 ± 10.9	68.0 ± 12.3	66.7 ± 10.7	0.08
BW (kg)	61.8 ± 13.5^‡^	61.4 ± 12.5^‡^	65.6 ± 13.0^*†^	<0.0001
BMI	24.6 ± 4.1	24.6 ± 5.5	24.9 ± 3.8	0.64
History of hypertension (years)	7.3 ± 5.9	no data	10.3 ± 5.8	<0.001
	(n = 430)		(n = 226)	
Current smoker	102 (26%)	152 (21%)	39 (10%)	<0.001
	(n = 392)^‡^	(n = 730)^‡^	(n = 380)^*†^	
Current drinker	186 (51%)	290 (45%)	200 (52%)	0.04
	(n = 366)	(n = 646)	(n = 382)	
DM	85 (17%)^‡^	146 (15%)^‡^	306 (54%)^*†^	<0.001
IHD	7 (1%)^†‡^	44 (5%)^*^	24 (6%)	<0.001
			(n = 375)^*^	
CVD	15 (3%)	33 (4%)	14 (4%)	0.85
			(n = 371)	
eGFR (mL/min/1.73m^2^)	80.3 ± 19.0	no data	75.4 ± 11.3	<0.001
	(n = 488)		(n = 385)	
Presence of proteinuria or albuminuria	0	0	0	
Estimated salt intake (g/day)	no data	no data	9.5 ± 2.1	
			(n = 91)	
Presence of the guidance of salt restriction diet	392 (80%)	no data	265 (74%)	0.04
Numbers of antihypertensive drugs	1.9 ± 0.9^‡^	2.0 ± 0.9^‡^	1.6 ± 0.8^*†^	<0.001
The prescription of antihypertensive drugs				
CCB	390 (80%)^‡^	713 (75%)^‡^	250 (65%)^*†^	<0.001
ARB	360 (74%)	742 (78%)^‡^	261 (68%)^†^	<0.001
ACEi	27 (6%)^‡^	38 (4%)	8 (2%)^*^	0.04
MRB	13 (3%)^†‡^	69 (7%)^*^	24 (6%)^*^	0.002
β-blocker	55 (11%)	87 (9%)	37 (10%)	0.45
α-blocker	37 (8%)^‡^	76 (8%)^‡^	11 (3%)^*†^	0.002
Diuretics	56 (12%)^†^	116 (12%)^*‡^	21 (6%)^†^	<0.001
Thiazide	52 (11%)	113 (12%)	no data	0.47
Loop	4 (1%)	3 (0.3%)	no data	0.20

♯P-values were analyzed by chi-square test for nominal data, or by ANOVA for continuous data on the comparison between the three studies. On the comparison between two studies, p-values were analyzed by chi-square test for nominal data or unpaired t-test for continuous data. Post hoc analysis with Bonferroni correction was performed when significancy was observed between three studies. The following symbols indicate the significant difference; * vs. 2011, ^†^ vs. 2014, and ^‡^ vs. 2021. ACEi: angiotensin converting enzyme inhibitor; ANOVA: analysis of variance; ARB: angiotensin Ⅱ receptor blocker; BMI: body mass index; BW: body weight; CCB: calcium channel blocker; CKD: chronic kidney disease; CVD: cerebral vascular disease; DM: diabetes mellitus; eGFR: estimated glomerular filtration rate; IHD: ischemic heart disease; MRB: mineralocorticoid receptor blocker.

Regarding patients in the CKD (+) group, our 2021 study included patients with larger BW and BMI, and higher incidence rates for complications such as diabetes and the presence of proteinuria or albuminuria than those in the 2011 and 2014 studies (2021 vs. 2011, p < 0.001, 0.01, <0001, and <0.001, respectively; 2021 vs. 2014, p = 0.001, 0.01, <0001, and <0.001, respectively). Regarding the prescription of antihypertensive drugs, a significantly reduced number of antihypertensive drugs were prescribed in the 2021 study (1.8 ± 0.9) compared to the 2011 and 2014 studies (p = 0.007 and <0.001, respectively).

A significantly lower frequency of prescription of angiotensin Ⅱ receptor blockers (ARBs), α-blocker, and diuretics in the 2021 study was observed than that reported in our 2011 and 2014 studies (p < 0.001 for all comparisons).

As reported in our 2021 study, the patient characteristics trend for patients without CKD was similar to those with CKD (Table 1b). Further, the reduced numbers of women and the utilization of angiotensin-converting enzyme inhibitor and calcium channel blocker (CCB) were shown in the 2021 study (p = 0.04, 0.04, and <0.001, respectively).

### Adjusted blood pressure in the office and at home recorded in each study

BP in the office and at-home measurements and the formula used to calculate the adjusted BP values for age, sex, BMI, the existence of diabetes, current drinking activity, and current smoking status are shown in Table 2. After adjustment, the CKD (+) group displayed significantly reduced office diastolic BP, home systolic BP, and home diastolic BP levels compared to those reported in 2011 (p = 0.03, <0.001, and 0.03, respectively). Compared to the 2011 and 2014 studies for the CKD (−) group, a significantly lower level of systolic home BP was observed in the 2021 study (p < 0.001, respectively).

**Table 2.** Office and Home BP Values Stratified by Target BP Values in Each Study Year.

Study year			2011	2014	2021	p Value by ANOVA	Post-hoc analysis by Bonferroni correction
Unadjusted data							
CKD (+)	Number of cases		316	152	396		
	Number of cases who measured home-BP		178	145	204		
	Office-BP	SBP	132.9 ± 13.4	135.6 ± 17.3	134.9 ± 17.1	0.14	
		DBP	74.6 ± 9.6	75.4 ± 12.2	74.8 ± 11.5	0.74	
	Home-BP	SBP	129.8 ± 12.3	132.2 ± 11.8	127.6 ± 8.0	<0.001	2014 vs. 2021, p < 0.001
		DBP	75.1 ± 9.3	75.8 ± 10.5	75.0 ± 8.4	0.68	
CKD (−)	Number of cases		488	946	385		
	Number of cases who measured home-BP		268	896	269		
	Office-BP	SBP	133.2 ± 12.8	132.7 ± 14.0	138.0 ± 17.4	<0.001	2011 vs. 2021, p < 0.001,
							2014 vs 2021, p < 0.001
		DBP	76.7 ± 9.0	77.5 ± 10.4	78.9 ± 11.1	0.006	2011 vs. 2021, p = 0.005
	Home-BP	SBP	127.7 ± 9.6	129.0 ± 9.9	128.7 ± 9.5	0.14	
		DBP	75.8 ± 8.3	76.3 ± 8.6	77.7 ± 7.9	0.02	2011 vs. 2021, p = 0.02,
							2014 vs. 2021, p = 0.04
Adjusted with age, sex, BMI, the existence of DM, current smoker, and current drinker							
CKD (+)	Office-BP	SBP	133.96 ± 1.07	133.68 ± 1.21	133.91 ± 0.81	0.07	
		DBP	75.53 ± 3.26	75.21 ± 3.97	74.76 ± 3.72	0.04	2011 vs. 2021, p = 0.03
	Home-BP	SBP	129.19 ± 1.89	129.42 ± 1.74	128.61 ± 1.30	<0.001	2011 vs. 2021, p < 0.001,
							2014 vs. 2021, p < 0.001
		DBP	74.72 ± 3.05	74.61 ± 3.76	74.01 ± 3.33	0.03	2011 vs. 2021, p = 0.03
CKD (−)	Office-BP	SBP	133.85 ± 1.07	134.00 ± 0.98	133.94 ± 0.81	0.06	
		DBP	77.79 ± 3.62	77.51 ± 4.26	77.31 ± 4.00	0.26	
	Home-BP	SBP	128.88 ± 1.78	128.76 ± 1.68	128.36 ± 1.30	<0.001	2011 vs. 2021, p < 0.001,
							2014 vs. 2021, p < 0.001
		DBP	76.74 ± 3.37	76.37 ± 3.74	76.16 ± 3.54	0.08	

Adjusted with age, sex (male = 0, female = 1), BMI, DM (with = 1, without = 0), CKD (with = 1, without = 0), smoker (current = 1, no or past = 0), and drinker (current = 1, no or past = 0) Office-SBP = 133.061+Age*0.021+Sex*0.689-BMI*0.022-DM*0.435-Current smoker*1.902+Current drinker*0.274 Office-DBP = 100.948-Age*0.306-Sex*1.474-BMI*0.046-DM*2.837-Current smoker*2.893+Current drinker*0.740 Home-SBP = 122.823+Age*0.062-Sex*0.344+BMI*0.029-DM*0.272+ Current smoker*3.004+Current drinker*1.273 Home-DBP = 94.554-Age*0.273-Sex*1.291+BMI*0.049-DM*2.147-Current smoker*0.540+Current drinker*0.723 ANOVA: analysis of variance; CKD: chronic kidney disease; DBP: diastolic blood pressure; DM: diabetes mellitus; SBP: systolic blood pressure.

### Blood pressure targets and their associated achievement rates

Supplementary Figure 4 shows the achievement rates for the office BP target of <140/90 mmHg and the home BP target of <135/85 mmHg. In the 2021 study, comparing the three studies, the CKD (+) group displayed a significantly high achievement rate for a home BP target of <135/85 mmHg (p < 0.05). In contrast, the achievement rate for an office BP target of <140/90 mmHg in the 2021 study was significantly reduced than in the 2011 study (p < 0.05). For the CKD (−) group, the achievement rate for an office BP target of <140/90 mmHg was significantly reduced in the 2021 study compared to the 2011 and 2014 studies (p < 0.05, respectively). A comparison between the CKD (+) and CKD (−) groups revealed significant differences in office and home BP levels in the 2014 study (p = 0.02, and 0.005, respectively).

**Figure 1.**
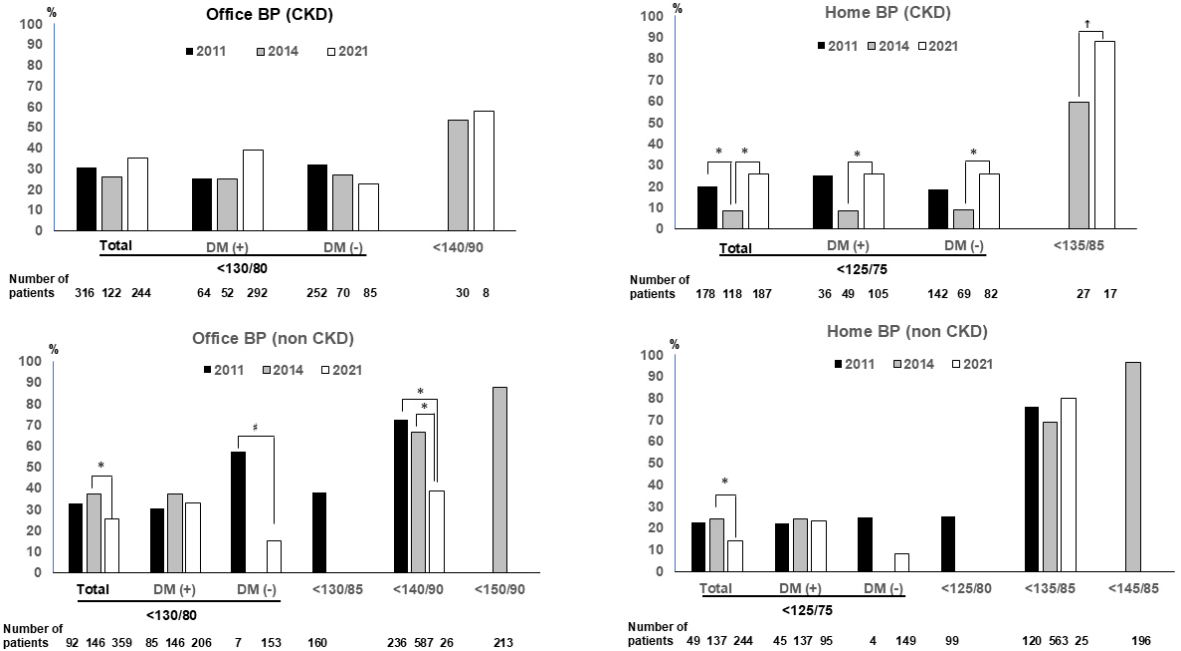
Achievement rate for a target office and home BP in accordance with JSH guidelines. BP: blood pressure; CKD: chronic kidney disease; DM: diabetes mellitus; JSH: Japanese Society of Hypertension.

Figure 1 shows the achievement rates for office and home BP targets in light of the JSH2019 guidelines. Significant differences in achievement rates for an office BP target were not observed. However, the achievement rates for a home BP target showed significant improvement in the CKD (+) group of patients compared to the 2014 study (p < 0.05 in patients with a home BP target of <125/75 mmHg, and p = 0.05 in those with a home BP target of <135/85 mmHg). In contrast, the achievement rates for patients in the CKD (-) group with a stringent BP target were significantly reduced in the 2021 study than in the 2014 study (p < 0.05) and this difference was notable in patients without diabetes (p = 0.004). A significant reduction in achievement rates for an office BP target of <140/90 mmHg was also observed in CKD (-) group patients (p < 0.05). Comparisons between the CKD (+) and CKD (−) groups for each study year are shown in Supplementary Figure 5. Compared to patients in the CKD (−) group, significantly reduced achievement rates for a home BP target were observed in the CKD (+) group in the 2014 study (p < 0.001). However, significantly higher achievement rates for both office and home BP targets were observed in the CKD (+) group compared to the CKD (−) group in the 2021 study (p = 0.003 and p = 0.002, respectively).

Supplementary Table 1 demonstrated the first and second BP values in the three studies. In the 2011 and 2014 studies, the second office BP values were required in principle, and the missing data rate was 0.8% and 0.1%, respectively. In contrast, in the 2022 J-DOME study, the second office BP values were required but not compulsory, and the second office BP values were recorded in 300 patients (36%). Significantly lower systolic BP was observed at the second measurement than the first one in the 2021 study (p < 0.001).

### Types and number of antihypertensive drugs in the 2021 study

In the 2021 study, the breakdown for the number of antihypertensive drugs prescribed per patient in the CKD (+)/CKD (−) groups was as follows: none, n = 23 (6%)/24 (6%); one drug, n = 115 (29%)/159 (41%); two drugs, n = 197 (50%)/156 (41%); three drugs, = 43 (12%)/36 (9%); four drugs, n = 11 (3%)/6 (2%); five drugs, n = 1 (0.3%)/1 (0.3%), respectively.

**Figure 2.**
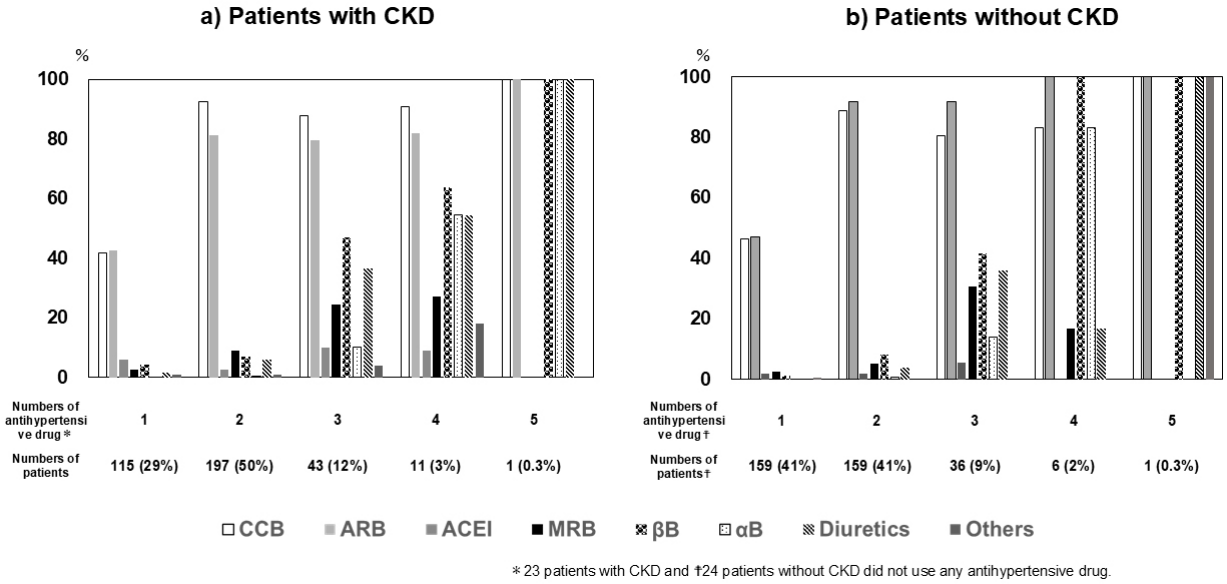
Frequency of the use of antihypertensive drugs stratified according to the number of drugs used in the 2021 study. ACEi: angiotensin-converting enzyme inhibitor; ARB: angiotensin II receptor blocker; CCB: calcium channel blocker; MRB: mineralocorticoid receptor blocker.

Figure 2 shows the frequency of use for various antihypertensive drugs, stratified according to the number of prescribed drugs. CCB or ARB drugs were most often prescribed in patients who took only one antihypertensive drug, whereas a combination of CCB and ARB was most often used in patients with two drugs. The use of diuretics was limited to the treatment of patients with administration of three or more antihypertensive drugs. These trends were common to both CKD (+) and CKD (−) patient groups.

## Discussion

This study clarified the status of BP management in patients with and without CKD in clinical practice in Japan. Patients with CKD and assigned a stringent BP target had significantly improved home BP measurements in the 2021 study compared to the 2014 study; however, it could not be said that they achieved their BP goals sufficiently. In contrast, the achievement rates of stringent office and home BP targets in patients without CKD were also low; moreover, these target achievement rates declined significantly in the 2021 study compared with the 2014 study. Notably, among patients with a stringent BP target, those without CKD had a significantly lower achievement rate in the 2021 study, a trend that is opposed to the one seen in our previous studies. Despite the low achievement rates for BP targets, a significant decline in the number of concomitant antihypertensive drugs prescribed was observed in the 2021study that involved a reduced frequency in the use of diuretics that are recommended in the event of poor BP control, as opposed to the administration of a combination treatment comprised of ARB and CCB.

The BP target was determined individually, depending on the characteristics or complications of each patient, and many guidelines have recommended more stringent BP management, namely setting a BP target of <130/80 mmHg for many patients with hypertension ^(1), (16), (17)^. Our previous study, where we recalculated the achievement rate individually, depending on the BP target assigned, showed low achievement rates (30.4% for an office BP target of <130/80 mmHg and 19.1% for a home BP target of <125/75 mmHg) ^(7)^.

According to the JSH2019 guidelines, most patients with CKD require an office BP target of <130/80 mmHg, except for those with CKD but without diabetes or proteinuria and more than 75 years of age ^(1)^. Moreover, during the course of the SPRINT clinical trial that required a BP target of 120/80 mmHg for patients in the intensive treatment group, the mean systolic BP was greater than 123 mmHg in patients with CKD, compared to 119 mmHg in patients without CKD ^(18), (19)^. These findings highlight the difficulty of BP control in patients with CKD compared to those without CKD. Based on these findings, the control rate in patients with CKD is suspected to be lower than that reported in the abovementioned studies that included all patients with hypertension. However, the control rate in patients with hypertension and CKD has not been reported.

According to a 20-year-old report, among 3,213 patients with CKD, 37% (95% confidence interval, 34.5% to 41.8%) showed BP <130/80 mm Hg ^(20)^. In a recent German CKD study, 49.3% of the patients with CKD and hypertension had controlled BP; however, the BP target was <140/90 mmHg ^(21)^. Regarding the Japanese reports of the achievement rates of a BP target in patients with CKD, Miyazawa et al. ^(11)^ reported on a CKD cohort (the Miyagi Gonryo CKD study) revealing that systolic BP/diastolic BP values were 131.5 mmHg/76.7 mmHg at the registered time, and the achievement rate for a BP target of <130/80 mmHg was 37%. A cross-sectional study of outpatients with CKD in Kyushu, Japan reported that 78.5% of elderly patients and 73.8% of young/middle-aged patients achieved a BP target of <140/90 mmHg, while those who achieved a target of <130/80 mmHg were only 29.6% and 22.4%, respectively ^(22)^. Yamagata et al. ^(23)^ conducted a prospective study enrolling patients with CKD who regularly visited their GPs (the From-J study) to encourage patients with CKD to consult a GP, enhance cooperation between nephrologists and GPs, and prevent kidney disease progression. Advanced intervention in CKD management showed no significant differences in systolic BP and diastolic BP at the minimal level during the study period (133 mmHg and 74 mmHg, respectively) ^(23)^, and these results suggest the modest achievement rates for a BP target of <130/80 mmHg in clinical practice in Japan.

One of the most noteworthy results of our 2021 study was the significant improvement in the achievement rate for a home BP target compared to our 2014 study. An extremely low achievement rate in the 2014 study that may have resulted from patient selection bias cannot be denied; however, CKD campaigns carried out all over Japan over the past 10 decades, as well as the revision of the JSH2014 that clearly stated that emphasis should be placed on managing home BP rather than office BP, might have influenced this improvement. Another possibility is that the achievement rate in patients without CKD decreased. Supplementary Table 2 compares the characteristics and details of the antihypertensive drugs used in patients with and without CKD in the 2021 study. Patients in the CKD (-) group were significantly young and had short periods of hypertension history compared to those in the CKD (+) group (p < 0.001). The prevalence of diabetes, IHD, CVD, and eGFR values was significantly lower in patients without CKD than in those with CKD, indicating that patients with CKD presented a higher risk of cardiovascular or renal outcomes than those without CKD. Therefore, intensive treatments for patients with CKD were administered, including a larger number of antihypertensive drugs, the use of CCB, the use of diuretics, the low prevalence of current drinkers, and the low estimated salt intake despite similar rates for the presence of salt restriction diet guidance compared to those without CKD (p = 0.002, 0.04, 0.02, 0.008, and 0.03, respectively). This led to a significant improvement in the achievement rate for a home BP target in patients with CKD in the 2021 study. Although BP management for young and middle-aged individuals who do not have risk factors for cardiovascular complications such as diabetes or CKD has been tightened to <130/80 mmHg in the JSH2019 guidelines, the achievement rate for the assigned BP target was particularly low in our previous study ^(7)^. It is important to carry out further campaigns for patients with CKD, who still show a relatively reduced BP control; however, in contrast, we need to be careful with people without CKD, especially young and middle-aged individuals. It is also necessary to educate patients without CKD about stringent BP control to prevent the onset of CKD or cardiovascular events.

### Study limitations

The most serious concern is the study participants in the 2021 study. Data from 26 medical hospitals or clinics were used, and the results in the 2021 study showed a significantly larger proportion of patients with diabetes than in the 2011 and 2014 studies (p < 0.001, respectively; Table 1). Furthermore, 94.6% of the patients had an office BP target of <130/80 mmHg which differed markedly from targets set in the 2011 and 2014 studies (Supplementary Figure 3). The revised JSH guidelines may have influenced the BP target levels. Such a large difference in BP targets leads to serious concerns about the acceptability of comparisons between our three studies. We cannot exclude the possibility that participants in the 2021 study may not be representative of Japanese participants with hypertension.

There are also some concerns about the method and frequency of BP measurements. In principle, BP measurement was performed by GPs in accordance with the JSH2019 guidelines, however, the BP measurement device or cuff sizes are not standardized among all medical facilities because a single device was not insisted on for each study. In addition, 64% of the second office BP values were missing in the 2021 data from J-DOME. There could be several reasons; e.g. GPs registered only first data to the database although multiple measurements were taken in the hospital, or GPs deliberately recorded the average BP value to the database. Significantly lower systolic BP was observed at the second measurement than the first one in the 2021 study (p < 0.001, Supplementary Table 1), suggesting that different results may be observed if data from multiple BP measurements are analyzed. Registry data in a larger sample size might have justified the ambiguity in BP measurement, and, in the future, accumulating more cases nationwide and further improving the reliability of the data in J-DOME will lead to more robust results.

In conclusion, unlike trends in patients without CKD, home BP control in patients with hypertension and CKD has improved over the past decade. However, the rate of achieving stringent BP targets in these patients remains unsatisfactory. This indicates a need for additional campaigns to raise awareness about CKD and to resolve the clinical inertia stemming from an insufficient number of concomitant drugs.

## Article Information

### Conflicts of Interest

Kei Asayama is an academic consultant for Omron Healthcare, Co, Ltd. The other authors declare that they have no conflicts of interest.

### Acknowledgement

We are grateful to all the participants of this study and acknowledge the support of the members of the Kanagawa Physicians Association who participated in our study. We also thank the physicians who participated in the JDOME study. Also, we especially thank Nobuo Hatori who greatly contributed to the statistical analysis in this study.

### Author Contributions

All authors read and approved the final manuscript. The main contributions of the authors for this manuscript are as follows: Kazuo Kobayashi, Keiichi Chin, Takayuki Furuki, Hiroyuki Sakai, Masaaki Miyakawa, Kei Asayama, Narumi Eguchi, Tomohiro Katsuya, Kazuyoshi Sato, Kouichi Tamura, and Akira Kanamori made the design of this study.

Masaaki Miyakawa, Kei Asayama, Narumi Eguchi, Tomohiro Katsuya, and Kazuyoshi Sato collected the data and built the dataset as J-DOME.

Kazuo Kobayashi, Keiichi Chin, Takayuki Furuki, Hiroyuki Sakai, Kazuyoshi Sato, and Kouichi Tamura Performed a statistical analysis of this manuscript.

Kazuo Kobayashi and Kouichi Tamura were major contributors in writing the manuscript.

### Approval by Institutional Review Board (IRB)

This study was approved by the Ethics Committee of the Kanagawa Prefecture Medical Association, Japan (Ethical Approval Number: KREC2204; July 29, 2022). The Ethical Approval of J-DOME was obtained through the ethics review board of the Japan Medical Association (Ethical Approval Number: 28-3; the latest approval was issued on February 17, 2023).

### Data Availability

The data can be used from the Kanagawa Physicians Association Data Access for researchers who are bound by confidentiality agreements.

Details for contact: Kanagawa Physicians Association, 3-1 Fujimicho naka-ku, Yokohama city, Kanagawa prefecture, Japan; e-mail: k-taishi@xc4.so-net.ne.jp (correspondence to Kazuo Kobayashi, MD, PhD).

### Disclaimer

Narumi Eguchi and Kouichi Tamura are the Editors of JMA Journal and on the journal’s Editorial Staff. They were not involved in the editorial evaluation or decision to accept this article for publication at all.

## Supplement

Supplementary Materials
