# Supplementary material for: Cross-sectional Survey of Hypertension Management among Patients Stratified by Chronic Kidney Disease in Japan
Source: JMA J. 2025 Aug 1;8(4):1240–9. doi: 10.31662/jmaj.2025-0037 (PMC12598252; doi:10.31662/jmaj.2025-0037)
Supplement: Supplementary Materials [file 2433-3298-8-4-1240-s001.pdf]

Supplementary Figure 1 Study participants

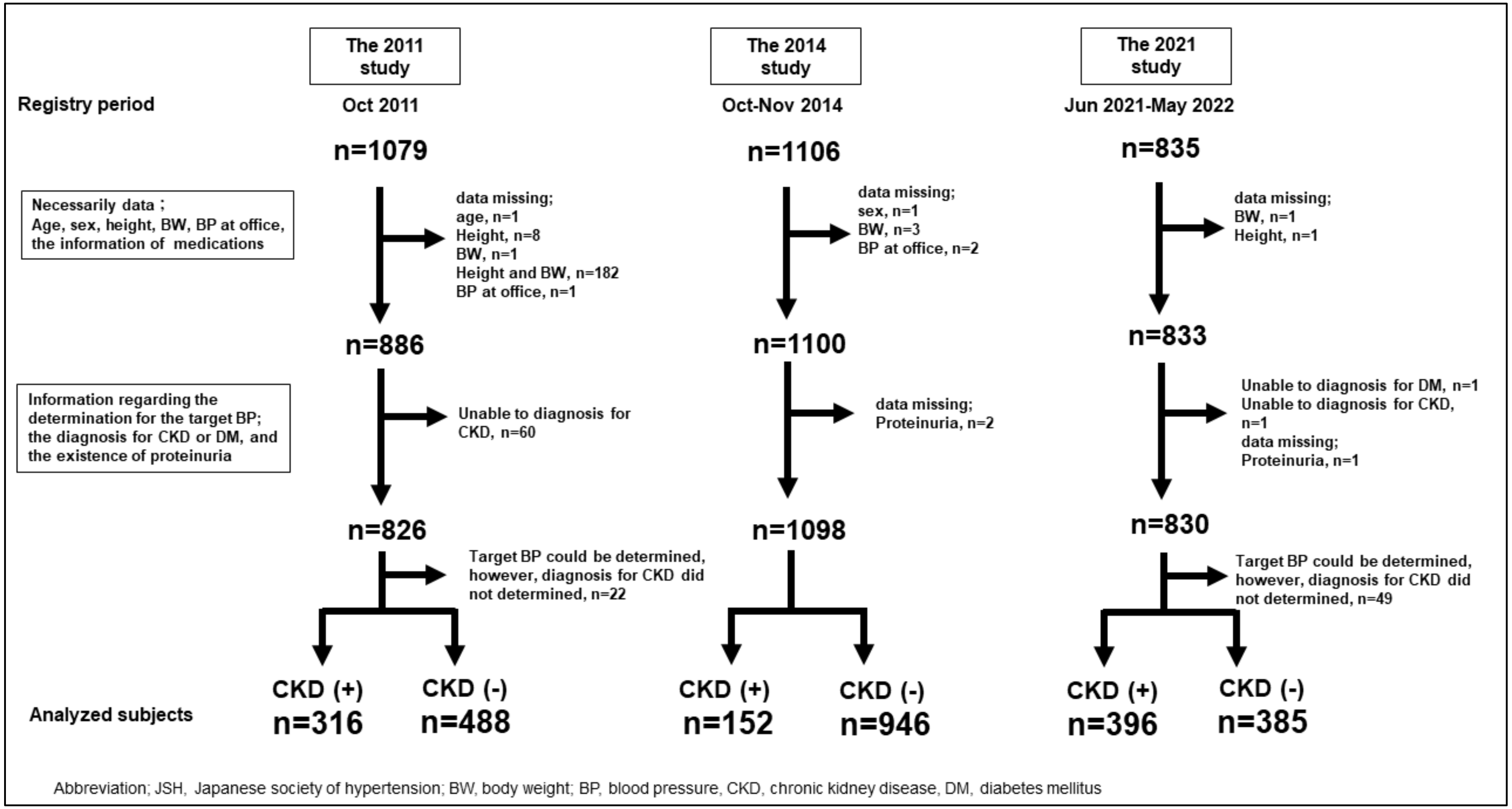

Supplementary Figure 2 Target office BP according to the JSH guidelines

<table>
<tr><th colspan="2"></th><th>JSH2009</th><th>JSH2014</th><th>JSH2019</th><th>mmHg</th></tr>
<tr><td colspan="2">DM</td><td>＜130/80</td><td>＜130/80</td><td>＜130/80</td><td></td></tr>
<tr><td rowspan="2">CKD</td><td>U-pro(＋)</td><td rowspan="2">＜130/80</td><td>＜130/80</td><td>＜130/80</td><td></td></tr>
<tr><td>U-Pro(－)</td><td>＜140/90</td><td>＜140/90</td><td></td></tr>
<tr><td rowspan="3">Age (y/o)</td><td>≥75</td><td rowspan="2">＜140/90</td><td>＜150/90</td><td>＜140/90</td><td></td></tr>
<tr><td>65≤ ＜75</td><td>＜140/90</td><td rowspan="2">＜130/80</td><td></td></tr>
<tr><td>＜65</td><td>＜130/85</td><td>＜140/90</td><td></td></tr>
<tr><td colspan="2">Cardiovascular disease</td><td>＜130/80
(after myocardial infarction)</td><td>＜140/90</td><td>＜130/80</td><td></td></tr>
<tr><td colspan="2">Cerebral vascular disease</td><td>＜140/90</td><td>＜140/90</td><td>＜130/80*</td><td></td></tr>
</table>

＊<140/90 mmHg for patients with cerebrovascular disease (bilateral carotid artery stenosis or cerebral main artery occlusion present or unevaluated)

The target home BP was defined as a BP value by subtracting 5 mmHg from the target office BP value.

Abbreviation; BP, blood pressure; CKD, chronic kidney disease; DM, diabetes mellitus; JSH, Japanese society of hypertension; U-pro, proteinuria

Supplementary Figure 3 Distribution of study subjects depending on the target BP

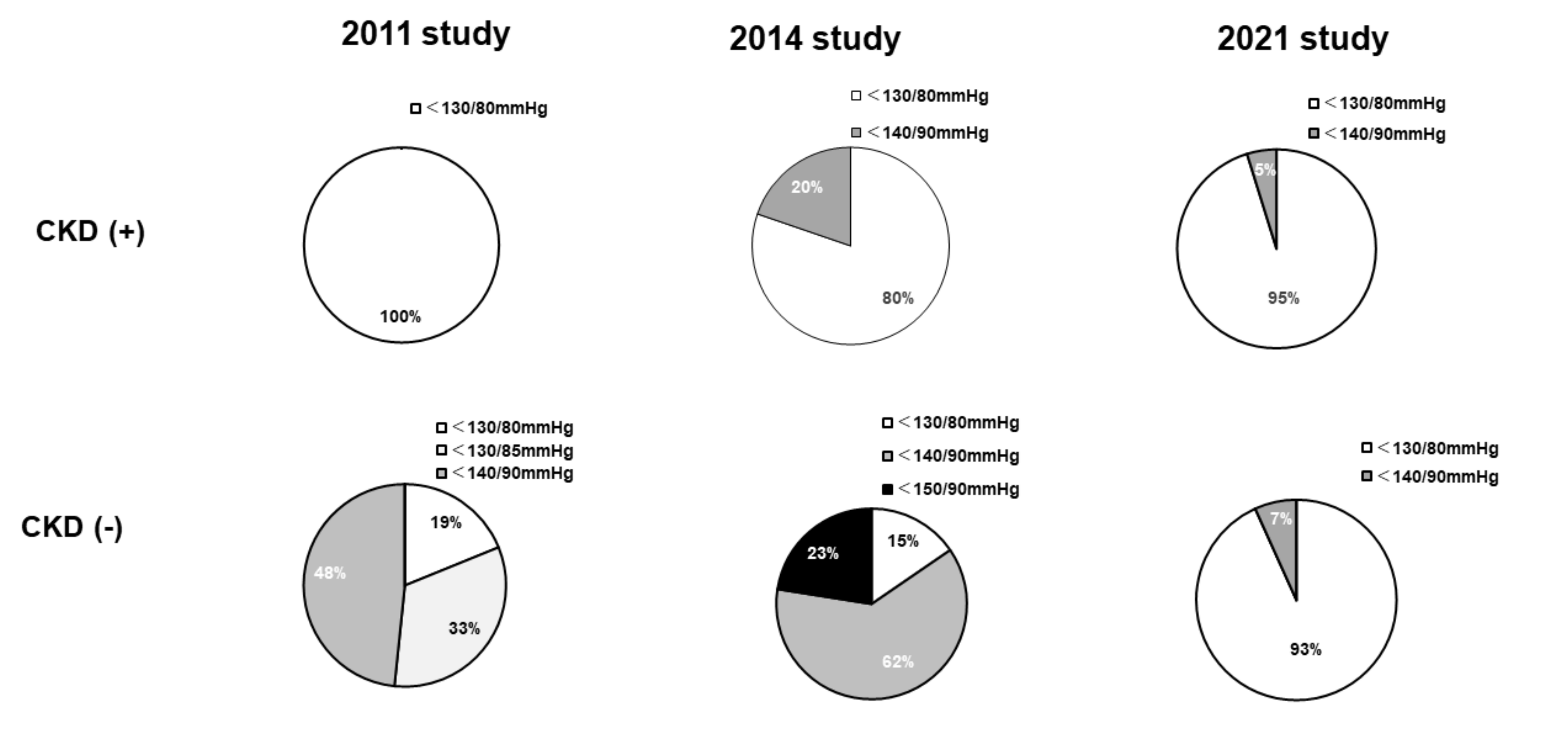

Abbreviation; BP, blood pressure; CKD, chronic kidney disease

Supplementary Figure 4 Achievement rates for the office BP target of <140/90 mmHg and home BP target of <135/85 mmHg

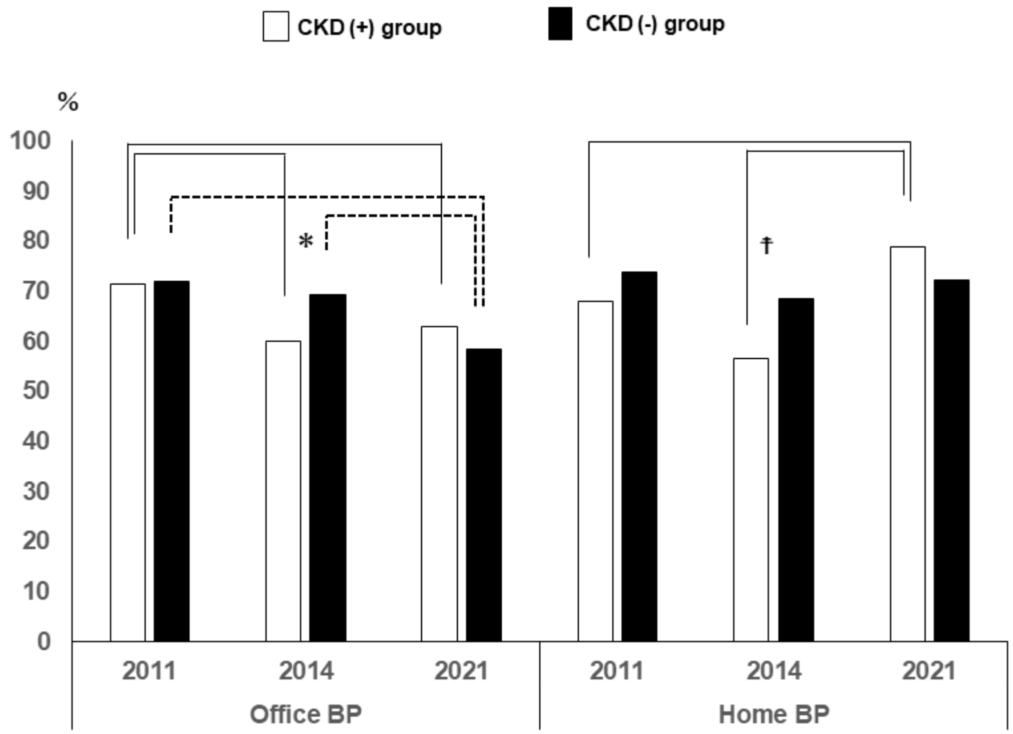

＊p=0.02 and †p=0.005 between CKD (+) and CKD (-) group by chi-square test

Solid line (CKD (+) group) and dotted line (CKD (-) group) showed p<0.05 by chi-square test with Bonferroni correction

Supplementary Figure 5 Achievement rates for the office BP target of 130/80 mmHg and home BP target of <125/75 mmHg

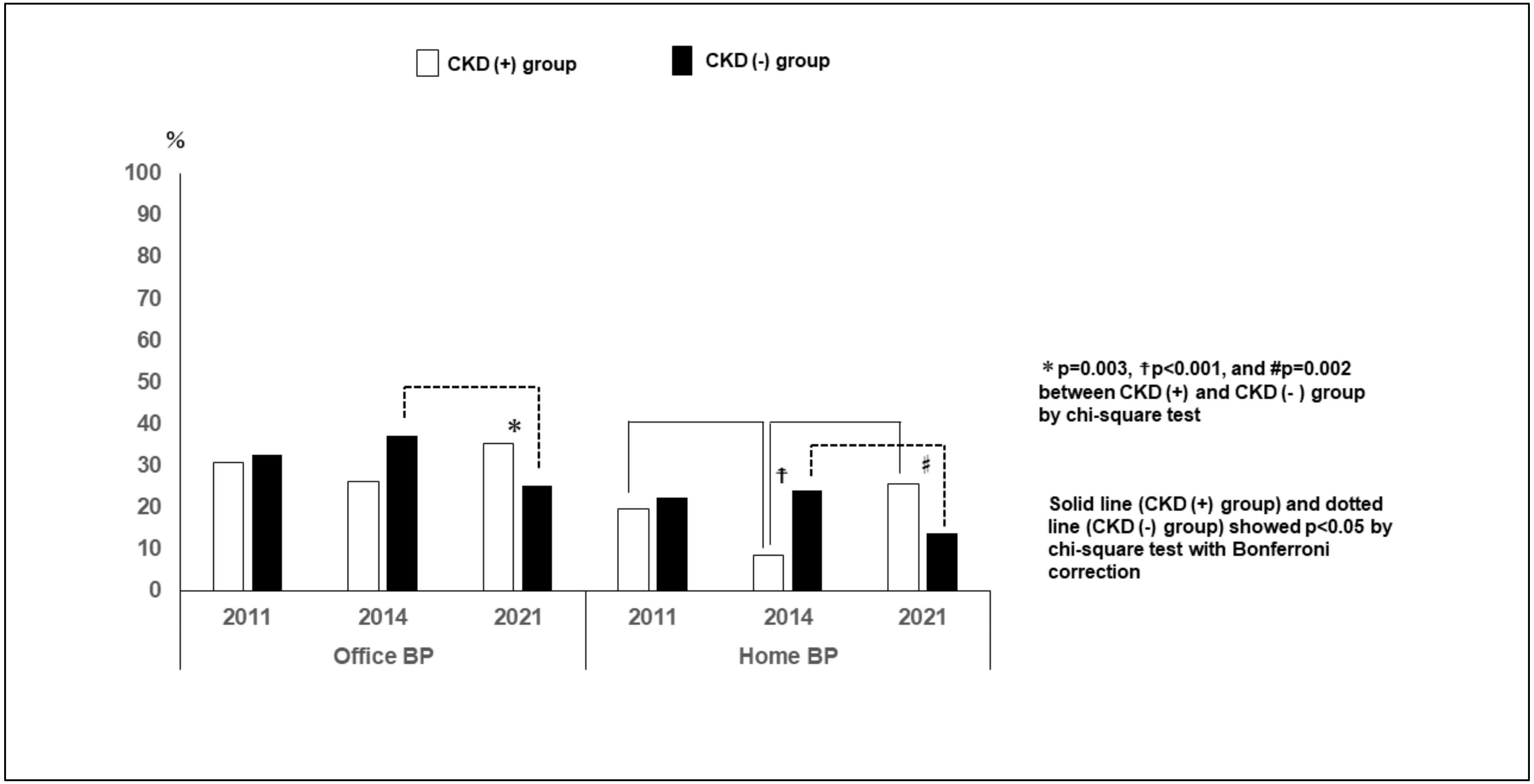

Supplementary Table 1. First and second BP values in the three studies

	Fist measurement	Second measurement	P-value*
The 2011 study			
Office systolic BP	133.0±13.0 (n=826)	132.4±13.2 (n=819)	0.12
Office second BP	75.8±9.3 (n=826)	75.6±9.5 (n=819)	0.50
The 2014 study			
Office systolic BP	133.1±14.6 (n=1098)	133.1±13.8 (n=1097)	0.86
Office diastolic BP	77.2±10.7 (n=1098)	77.2±10.4 (n=1097)	0.98
The 2021study			
Office systolic BP	141.3±18.2 (n=830)	136.5±22.5 (n=300)	<0.001
Office second BP	79.4±11.0 (n=830)	79.3±10.6 (n=283)	0.81

*paired t-test,
Abbreviation; BP, blood pressure

Supplementary Table 2. Characteristics and details of antihypertensive drugs of patients in 2021 study

	CKD (+)	CKD (-)	P-value
Number of cases	396	385	
Sex (women)	165 (42%)	176 (46%)	0.25*
Age (years-old)	72.9±10.2	66.7±10.7	<0.001†
BW (kg)	65.8±13.0	65.6±13.0	0.77††
BMI	25.3±3.6	24.9±3.8	0.11†
History of hypertension (years)	13.7±8.5 (n=237)	10.3±5.8 (n=226)	<0.001*
Current smoker	43 (11%) (n=396)	39 (10%) (n=380)	0.79*
Current drinker	168 (43%) (n=392)	200 (52%) (n=382)	0.008*
DM	292 (74%)	306 (54%)	<0.001**
IHD	57 (15%) (n=389)	24 (6%) (n=375)	<0.001**
CVD	36 (9%) (n=385)	14 (4%) (n=371)	0.002*
eGFR(mL/min/1.73m^2)	54.8±17.0 (n=395)	75.4±11.3 (n=385)	<0.001*
Estimated Salt intake (g/day)	8.9±2.0 (n=108)	9.5±2.1 (n=91)	0.03*
Presence of the guidance of salt restriction diet	269(76%)	265 (74%)	0.59*
Presence of proteinuria or albuminuria	135 (37%) (n=369)	0	<0.001*
Numbers of antihypertensive drugs	1.8±0.9	1.6±0.8	0.002*
The prescription of antihypertensive drugs			
CCB	284 (72%)	250 (65%)	0.04*
ARB	258 (65%)	261 (68%)	0.44*
ACEi	18 (5%)	8 (2%)	0.06*
MRB	36 (9%)	24 (6%)	0.13*
β-blocker	50 (13%)	37 (10%)	0.18*
α-blocker	13 (3%)	11 (3%)	0.73*
Diuretics	39 (10%)	21 (6%)	0.02*

*Chi-square test, † unpaired t-test,

Abbreviation; ACEi, angiotensin converting enzyme inhibitor; ANOVA, analysis of variance; ARB, angiotensin Ⅱ receptor blocker; BMI, body mass index; BW, body weight; CCB, calcium channel blocker; CKD, chronic kidney disease; CVD, cerebral vascular disease; DM, diabetes mellitus; eGFR, estimated glomerular filtration rate; IHD, ischemic heart disease; MRB, mineralocorticoid receptor blocker
